# Action of combined magnetic fields on aqueous solution of glutamic acid: the further development of investigations

**DOI:** 10.1186/1477-044X-6-1

**Published:** 2008-01-25

**Authors:** Livio Giuliani, Settimio Grimaldi, Antonella Lisi, Enrico D'Emilia, Natalia Bobkova, Mikhail Zhadin

**Affiliations:** 1 Institute of for Prevention and Labor Safety (ISPESL), Rome, Italy; 2 Institute of Neurobiology and Molecular Medicine, CNR, Rome, Italy; 3 Institute of Cell Biophysics, Pushchino, Moscow Region, Russia

## Abstract

In the present work the results of the known investigation of the influence of combined static (40 *μT*) and alternating (amplitude of 40 *nT*) parallel magnetic fields on the current through the aqueous solution of glutamic acid, were successfully replicated. Fourteen experiments were carried out by the application of the combined magnetic fields to the solution placed into a Plexiglas reaction vessel at application of static voltage to golden electrodes placed into the solution. Six experiments were carried out by the application of the combined magnetic fields to the solution placed in a Plexiglas reaction vessel, without electrodes, within an electric field, generated by means of a capacitor at the voltage of 27 mV. The frequency of the alternating field was scanned within the bounds of 1.0 *Hz *including the cyclotron frequency corresponding to a glutamic acid ion and to the applied static magnetic field. In this study the prominent peaks with half-width of ~0.5 *Hz *and with different heights (till 80 *nA*) were registered at the alternating magnetic field frequency equal to the cyclotron frequency (4.2 *Hz*). The general reproducibility of the investigated effects was 70% among the all solutions studied by us and they arose usually after 40–60 min. after preparation of the solution. In some made-up solutions the appearance of instability in the registered current was noted in 30–45 min after the solution preparation. This instability endured for 20–40 min. At the end of such instability period the effects of combined fields action appeared practically every time. The possible mechanisms of revealed effects were discussed on the basis of modern quantum electrodynamics.

## Background

The middle of the eighties was marked with the discovery by Blackman [1,2] of a surprising phenomenon: a low frequency alternating (AC) magnetic field (MF) changed free calcium concentration in nervous tissue only in the presence of a simultaneously acting static (DC) MF. The most prominent effect was observed at the AC field frequency close to the cyclotron frequency of a calcium ion. The cyclotron frequency is defined [2] as

$$f_{C}=\frac{q}{2\pi m}B_{\text{o}},$$

where *q *and *m *are the charge and mass of the ion, and *B*_o _is the DC field. These works opened a new line of research in Bioelectromagnetics.

There were three unexpected qualities in this phenomenon: 1) the necessity of simultaneous action of DC and AC MFs, 2) the resonance effect on cyclotron frequency, and 3) very small values of acting MFs, measured with tens of *μT*, and extremely low frequencies of AC MFs, measured with several tens of *Hz*. Therefore, these results evoked a suspicion in the scientific community [3]. Afterwards, many confirmations for these data were obtained in works performed on different objects and in different experimental situations [4,5,5,7-13] which captured the interest of the scientific community about the existence of the above effects.

In the middle of the nineties a series of experiments were made, on aqueous solutions of amino acids. At the cyclotron frequencies measured by several *Hz*, which corresponded to the investigated amino acid ions, and at superweak AC MFs measured by tens of *nT*, the short-term increase in the current caused by application of the voltage to the investigated solution was revealed. These results were published in Russian journal "Biophysics" [14]. Afterwards the experiment and results described in the above article were successfully replicated in Italy [14,15] and in Germany [16]. These works confirmed the existence of the above effects of combined MFs, but the reproducibility of these experimental phenomena (~20%) was noticeably lower than ones in the original work. The present investigation was undertaken with the purposes of examining both the reproducibility and explaining the experimental effects of the action of combined magnetic field effect on aqueous solutions of glutamic acid.

## Methods

### 1. Glutamic acid solutions in the electrolytic cell

The experimental installation for investigation of MFs action on ionic conductance in glutamic acid solution in the electrolytic cell was made by means of a coil generating parallel DC and AC MFs inside itself, of the plexiglass cubic cell (8.0 *ml *in volume), in which the aqueous solution of glutamic acid was poured for investigation.

**Figure 1:**
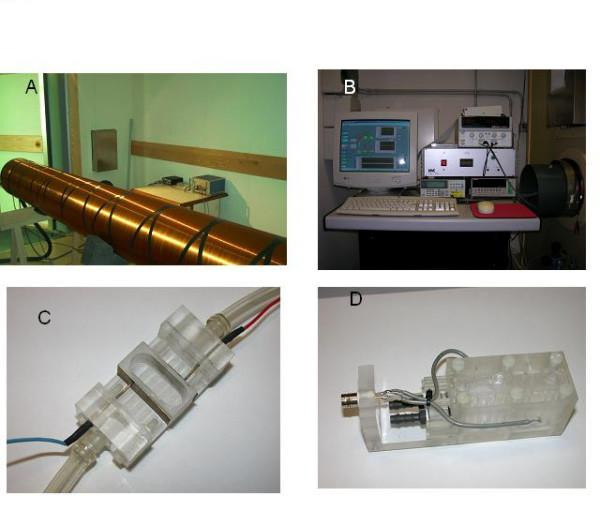
**Exposure Systems**. A – Solenoid in the magnetic shielded room, B – Power Supply and Remote Control consol, C – Reaction vessel with external electrodes, D – Reaction vessel with internal electrodes.

The coil axis was located horizontally. The cell was placed in the central part of the coil. Inside the cell two parallel plain squared golden electrodes of 20 *mm*^2 ^were placed, being sunken into the investigated solution. The distance between the two electrodes was 10 *mm*. The axis between centers of these electrodes was situated horizontally, being perpendicular to the coil axis. The potential difference between these two electrodes was 80.0 *mV *and was created by the accumulator. The current through the solution was about several *nA*. After appropriate amplification the current entered the measuring instrument was registered by the computer. Another accumulator also provided direct current through the coil for creating DC MF in the space inside this coil. The computer generated AC current through the digital-to-analog converter for the coil, creating AC MF with proper frequency. The coil with the cell was located in the special large Permalloy heat-insulated chamber shielding them from the influence of external MFs with a coefficient of shielding about 200 (Fig. 1). The computer and accumulator as well as the experimenters themselves were located outside the chamber during the experiment. The cell was heat controlled by means of forced circulating water, power outside of the chamber. The general views of the experimental installation inside and outside the chamber are shown in Fig. 1a and Fig. 1b respectively.

The glutamic acid solution (Sigma, USA; 0.38 *mg*/*ml*) was prepared in distilled water; the pH was adjusted to 2.89 by adding dilute hydrochloric acid solution at a temperature of 22°C. The experiment started right away after the solution was prepared. The DC MF along the coil axis was equal to 40.0 *μT *and the amplitude of AC MF was equal to 40.0 *nT*. The cyclotron frequency was equal to 4.2 *Hz *according to the charge-to-mass of glutamic acid ion and to DC MF used. The experiment process was computer controlled by a software performed scanning at the AC MF frequency in the range between 3 and 5 Hz including the cyclotron frequency of glutamic acid. The real duration of each AC frequency step exposure during scanning was 1,5 *s *that provided sufficient time for development of resonance. The total number of AC frequencies steps during one scanning was 200 steps each one of 0.01 Hz. The curve representing the dependence of current through the solution in relation to the frequency of exposed AC MF, was shown on the computer screen for each scanning.

### 2. Glutamic acid solutions in plexiglass reaction vessel

For investigation of MFs action on ionic conductance in glutamic acid solution in plexiglass reaction vessel, with electrodes not sunken in the solution, but placed each one at the distance of 13.5 mm, in a fashion to form a condenser at the voltage of 27 mV providing a DC electric field of 20 V/m, the same procedure was followed. The reaction vessel, built with the same dimensions of the electrolytic cell, was heat controlled as above and the arising current, at the cyclotron frequency, was detected by means of a coil (2.300 turns of 0.2 mm thick copper wires) surrounding the vessel. The induced signal in the coil was 10,000 times amplified.

## Experimental results

### 1. Glutamic acid solution in the electrolytic cell

**Figure 2:**
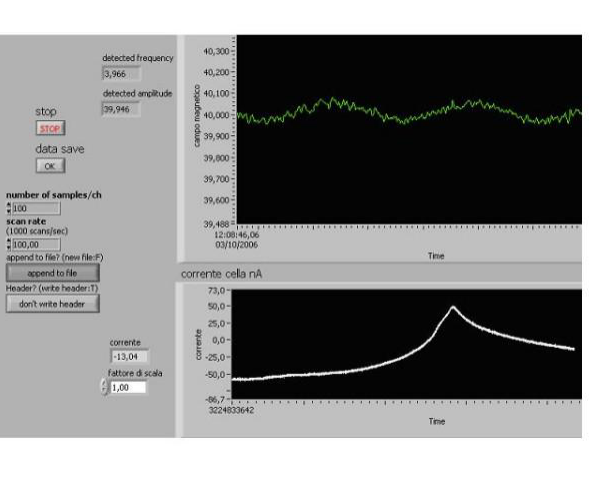
**The usual resonance curve**. Cell with gold electrodes and polarization 80 mV, Temperature : 22°C, L- glutamic acid 38 mg/ml, pH 2.89, B: 40 μT, B_o _: 40 nT, Sweep time: 100 sec, Total data current recorded :100 sec, Initial current: – 60 nA, Final current : – 10 nA, Scan rate :100 point/sec, Peak resonance frequency 3.966 Hz. The resonance peak with maximum near the cyclotron frequency of about 4.0 *Hz*. The abscissa: the scanning of frequency of alternating magnetic field. The ordinate: the current through the amino acid aqueous solution.

At the above described experimental conditions the usual form of the resonance peak represented the asymmetric resonance curve with the maximum close to the cyclotron frequency of AC MF (~4.2 *Hz*). These usual peaks had the rise half-width on the left of about 0.15 *Hz *and the slope half-width on the right of about 0.35 *Hz*, and the height of about 35 *nA *over the background current (Fig. 2).

Only in one case erratic peaks located outside immediate proximity to the cyclotron frequency arose at the exposition of the above combined DC and AC MFs. Right away after preparation of the solution there was not any expected effects during the scanning. The peaks arose in 40–60 *min *after preparation of the solution. As a rule, the once appeared effect could be repeated in the following scanning of AC MF frequency. The effects ceased to arise in about 60 *min *after its first arising, but sometimes it could be restored again after washout of electrode surfaces. As a whole, the average probability of effect arising was about 40% of performed scanning of different solutions.

**Figure 3:**
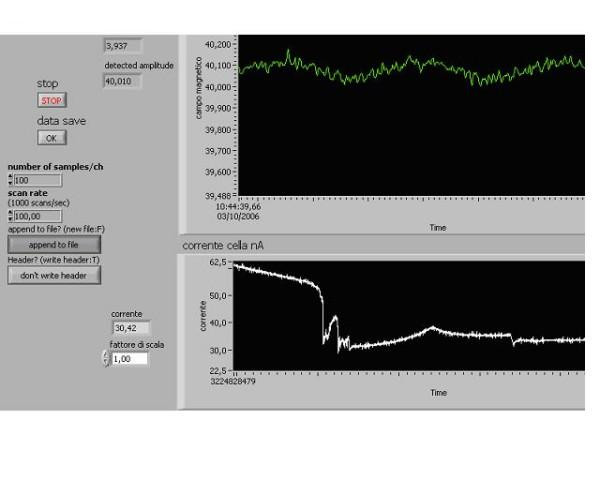
**The instability of the current through the solution**. Cell with gold washed electrodes and polarization 80 mV (washed with sulfonitric solution). Temperature : 22°C, L-glutamic acid, saturated solution with crystals on the bottom: pH 2.89. B: 40 μT, B_o _: 40 nT, Sweep time: 100 sec, Total data current recorded :100 sec, Initial current : 62,5 nA. Final current : 33 nA, Scan rate :100 point/sec, Peak resonance frequency 3,937 Hz. The abscissa: the time. The ordinate: the current through the amino acid aqueous solution.

In about 30% of the investigated solutions we observed the following rather unexpected phenomena. Approximately in 30–45 *min *after preparation of the new solution and beginning the experiment, the current through the solution spontaneously became to be unstable and revealed slow changes of several tens of *nA *(Fig. 3).

**Figure 4:**
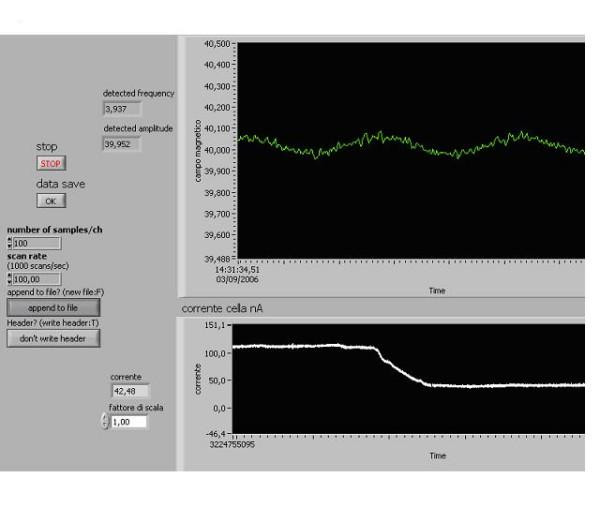
**The incomplete negative resonance curve**. Cell with gold electrodes and polarization 80 mV, Temperature : 22°C. L- glutamic acid, saturated solution with crystals on the bottom: pH 2.89, B: 40 μT, B_o _: 40 nT. Sweep time: 200 sec, Total data current recorded :531 sec, Initial current : 112 nA, Final current : 42,48 nA. Scan rate :100 point/sec, Resonance Frequency: 3.937 Hz. The decrease in the current through the solution at the frequency near the cyclotron frequency. The abscissa: the scanning of frequency of alternating magnetic field. The ordinate: the current through the amino acid aqueous solution.

Such sort of instability endured for about 20–40 *min*. If in about 15–25 *min *after its arising the above MFs were exposed and the scanning was switched on, the effect of this exposition arose nearly always – with probability of about 90%. Sometimes the effect arose in unusual form – in the form of quick change of the current value (Fig. 4).

Thus, on the background of such sort of instability the effects of combined MFs arose always in either one or another above described form.

### 2. Glutamic acid solution in plexiglass reaction vessel

**Figure 5:**
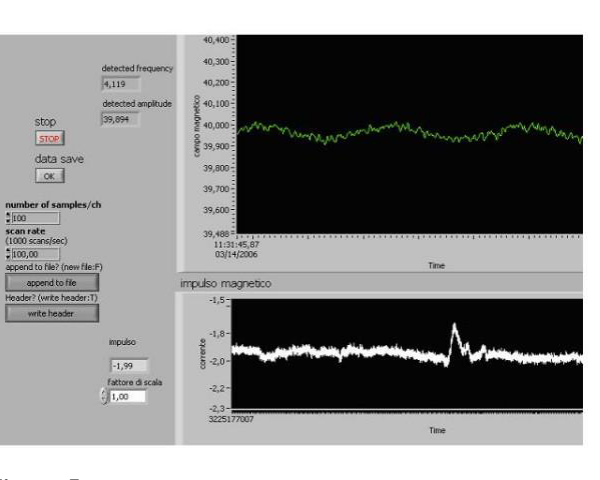
**The resonance curve in the experiment with electrodes outside the cell**. Cell with external electrodes and polarization 80 mV, Temperature : 22°C, L- glutamic acid 38 mg/ml, pH 2.89. B: 40 μT, B_o _: 40 nT, Sweep time: 100 sec, Total data current recorded :100 sec, Scan rate :100 point/sec, Peak resonance frequency 4,119 Hz. The resonance peak with maximum near the cyclotron frequency of about 4.0 *Hz*. The abscissa: the scanning of frequency of alternating magnetic field. The ordinate: the current in the coil detector.

In order to elucidate, whether the investigated phenomena are connected with the processes taking place in immediate proximity to the electrodes surfaces, as was supposed by Comisso et al. [16], we performed the following series of experiments. The electrodes were carried out of the cell and were pressed to the external surfaces of the cell walls. In this case, they only created the static electric field (20 V/m) inside the cell without closed electric circuit between the electrodes. Registration of electric phenomena inside the cell was made with the special coil detector located outside the cell, embracing it. It had 2300 turns and inductance of 158,6 *mH*. Exposition of DC and AC MFs caused the asymmetric peak with maximum at the cyclotron frequency of the glutamic acid ion (Fig. 5). The peak was shorter than the usual resonance peak in the experiments with electrodes inside the cell, and the asymmetry of the peak was more prominent. The reproducibility of the effect was slightly less then in the case of the electrolytic cell (67% instead of 73%).

## Discussion

The outcome of our present investigation confirmed the results of the original work [17] and of further works [18,16] devoted to replication of the original experiments: the height and half-width of the resonance peak of the current through the glutamic acid solution at the cyclotron frequency of AC MF were comparable with those in the above works. The total reproducibility of the expected effects in the present work (70%) was much higher then those (~20%) reported in these works [18,16]. Most likely it was due to longer duration of AC MF exposition applied in our work in every point of AC MF frequency as well as to the accuracy of the exposures provided in our facility in Rome, equipped with a patent pending exposure system, developed by Italian CNR and ISPESL institutions.

We were able to reveal a new effect. About 30% of made-up solutions in 30–45 *min *after their preparation manifested explicit (several tens of *nA) *temporary (about 20–40 *min *of duration) instability of the current through them (Fig. 3). If in the end of the period of such instability we started up the AC MF frequency scanning, the expected resonance effects arose every time: whether in the above described asymmetric resonance curve (70% of cases) or in the form of incomplete resonance curve as increase or decrease of the current (Fig. 4) at the vicinity of the cyclotron frequency of glutamic acid ion. So, at such instability in the effects of combined MFs at the cyclotron frequency arose always in either, one or another form. This instability of the current through the solution could be reliable sign of readiness of the solution for revealing the resonance effect on the action of combined DC and AC MFs. Below we'll return to interpretation of this phenomenon.

Earlier there were attempts to understand the physical mechanisms of resonance action of combined MFs. A. Liboff [2,19], considering the motion of free ions under action of these MFs, supposed a mechanism similar to the one working in a living system like in a cyclotron. But this idea could be realized only in very large systems capable to include the long radius of ion rotation measured by meters. The idea [20,12]of participation of parametric resonance in such sort of effects was not very fruitful for still unknown necessary low frequency harmonic oscillator in living systems. The Larmor precession could not help in this situation, not being such an oscillator because of lack of restoring force with proper parameters. Moreover, both of the above theories required a fine vacuum within the bounds of ion motion. The theory [17,21] of combined action of DC and AC MFs on thermal motion of bound ions showed that the wide diversity of biological effects of such MFs would be only possible, if the damping coefficient of oscillation within a microvolume around the ions had been no more than the circular cyclotron frequencies of exposed ions. Really, in experiments with combined MFs [4] it was revealed that the half-width of resonance peaks was comparable with the circular cyclotron frequency of AC MF, or less than this frequency. In the present work the half-width of the resonance peak obtained by us was also less than the circular cyclotron frequency of AC MF applied. It could be considered as a good confirmation of the above theory, if such estimation of damping coefficient had corresponded to viscosity and damping processes in aqueous solutions. But unfortunately, it turned out to be essentially less than the estimations made on the basis of Physics of Continuous Media [22] that provoked distrust of some scientists concerning weak MFs biological effects. Of course, it would be possible to contest the competence of such estimations and to prove inapplicability of ideas and formulas of Mechanics of Continuous Media to the processes within a microvolume comprising a small number of molecules where the notion of "viscosity" itself loses its meaning. But it would lead to long unsuccessful debate. The situation with physical foundation of Bioelectromagnetics of weak MFs was rather difficult. Decision of this problem had come from Quantum Electrodynamics of Condensed Matter. For the last 15 years the physical ideas on water structure and on aqueous solutions have radically changed themselves under the influence of works by Italian outstanding physicist Giuliano Preparata.

According to Quantum Electro-Dynamical Theory by G.Preparata [23], the liquid water consists of two components: coherent and incoherent ones. The coherent component is contained within spherical so called "coherence domains" (CDs) where all molecules synchronously oscillate with the same phase. CDs are surrounded by the incoherent component where molecules oscillate with casual phases regarding each other. Diameters of CDs are measured by tenths of a micron, and at room temperature the total volume of domains is about 40% of the whole water media. The stability of these domains is great because the bond energy of water molecules within them is much more than the thermal noise energy. Within CDs the viscosity and oscillation damping of coherent water essentially differ from ones of incoherent water.

Del Giudice et al. [15] considered the motion of a glutamic acid ion inside CD under the influence of combined DC and AC MFs at the cyclotron frequency of this ion. They found that in spite of a huge radius of the orbit on which this ion would rotate if it have performed the free motion under the influence of MFs at room temperature, within CD it really has to move along the spherical border of CD, performing the internal reflectance without any friction and energy loss. At resonance action of the cyclotron frequency the ion is accelerated by the MFs, increasing its kinetic energy till its escape from CD. The glutamic acid ions leaving CDs cause the peak of the current through the solution.

In a recent work Zhadin and Giuliani [24]theoretically revealed the mechanism of capture of glutamic acid ions by CDs, the mechanism of formation of "mixed CDs" in which glutamic acid ions become to participate in the coherent oscillations on an equal rights with water molecules, and the cyclic mechanism of successive changing of different ionic forms of glutamic acid molecules. At pH~3 in aqueous solution the glutamic acid ions exist in the form of so called "zwitterions" – long dipole ions with the total charge equal to zero. Due to its big length the zwitterions have big dipole moments. After preparation of the solution the zwitterions gradually form many wide clusters in the solution. Simultaneously the formation of water CDs all over the solution is developing. In the places of zwitterion cluster location, the water CDs inevitably capture numerous groups of glutamic acid molecules in the zwitterion form. This process is promoted by similarity between some parts of spectra of water molecules and glutamic acid ones, that allows to enter glutamic acid molecules into the process of forming CDs where they could participate in coherent oscillations pari passu with water molecules and have the same high bond energy within CD. The authors of the above work named such sort of CDs as "mixed CDs". It is necessary to mark that by no means all amino acids and all other substances are able to form mixed CDs. It depends on the spectral mutual compatibility between this substance molecules and water molecules, as well as on physicochemical features of the aqueous solution.

Due to the high bond energy which is much more than the energy of hydrogen bonds, the hydration shells of zwitterions dissolve among coherent water molecules within mixed CDs. (By the way, it explains the problem of long standing: why the cyclotron frequency definition takes into account of ion mass without its heavy hydration shell and only such cyclotron frequency is effective in the experiments). The energy of coherent oscillations close to the energy of ionization of glutamic acid molecule transforms zwitterions into usual ions of glutamic acid that further increases its kinetic energy in accordance with the mechanism of Del Giudice et al. [15] under the influence of combined DC and AC MFs and leads to its final escape from mixed CDs into the surrounding incoherent water, when forming the resonance peak of current through the solution. Turned out to be again in the media with pH~3, the usual ions of glutamic acid move to zwitterion form and whereupon the solution comes to the initial state.

This pattern puts some idea into the mind that electric instability in the solution after its preparation is connected with the processes of formation of zwitterion clusters and mixed CDs in the solution. It also makes understandable, why resonance action of combined MFs arises into particular prominence namely at the end of the period of instability, when the total number of mixed CDs is maximal. The reasons of not full reproducibility of the effects and even their total lack at weakly prominent electric instability or its absence become clear up too.

In Experimental Results section we described various manifestations of MFs effects, different time periods of their arising after the moments of the solution preparation in different experiments up to the total absence of any effect in some experiments. It could be connected with gradual disappearance of mixed CDs in the experimental solution. It is also possible that all this uncertainty is connected with insufficient stabilization of temperature within solutions in the course of the experiment. The solutions were prepared at the same temperature and then poured into the cell within the heat-controlled chamber. However, the temperature of the investigated solution in the cell could change a little during all the experiment. It is quite possible that formation of mixed CDs is very sensitive to the solution temperature.

In the work by Comisso et al. [16] there was a suggestion about possible essential role of the processes developing in immediate proximity to the surface of the electrodes in formation of resonance effects. In connection with this, we performed a series of experiments with the electrodes carried out of the cell and pressed to the external surfaces of the cell walls. Using the special coil detector of the electric state of the solution in these experiments, we obtained the prominent resonance peaks denoting the formation of resonance peaks of the current occurring in more or less degree all over the whole volume of the solution. It is in a good agreement with the above described pattern of the development of MFs resonance effects.

The asymmetry of the resonance peaks obtained in all our experiments is, most likely, connected with comparatively slow processes in the development of current peaks within the incoherent component of the water medium and of reestablishment of the initial state of the solution. If this is the case, then at the back direction of scanning it would be possible to expect the inversion in the asymmetry of the resonance peak. It could be interesting to verify it in the further development of these investigations.

## Cocnclusion

A new way to detect the so called Zhadin effects has been successfully attempted: the solution has been placed in a reaction vessel without electrodes within a condenser at 27 mV, an equipment more suitable to be applied to samples of biological tissues taking into account even the weak magnetic field generated by ion currents in the cells.

Our findings replicate previous investigation [17] of the influence of combined static and alternating parallel magnetic fields on the current through the aqueous solution of glutamic acid; outlining the relevance of low frequency electro-magnetic field in interacting with biological target. In addition, our results demonstrate that at combined static and alternating parallel, magnetic fields matching the ion cyclotron energy resonance of a particular charged molecule into biological tissue an intrinsic weak magnetic field is generated by ion currents in the cell.

These results should increase the reliability and the clinical feasibility of the use of electromagnetic field, tuned at ion cyclotron resonance of charged molecules, as a biophysical approach to interfere with biological mechanisms.

## Competing interests

The author(s) declare that they have no competing interests.
